# Optical coherence tomography for identification and quantification of human airway wall layers

**DOI:** 10.1371/journal.pone.0184145

**Published:** 2017-10-05

**Authors:** Julia N. S. d’Hooghe, Annika W. M. Goorsenberg, Daniel M. de Bruin, Joris J. T. H. Roelofs, Jouke T. Annema, Peter I. Bonta

**Affiliations:** 1 Department of Pulmonology, Academic Medical Center, University of Amsterdam, Amsterdam, the Netherlands; 2 Department of Biomedical Engineering & Physics, Academic Medical Center, University of Amsterdam, Amsterdam, the Netherlands; 3 Department of Pathology, Academic Medical Center, University of Amsterdam, Amsterdam, the Netherlands; Simon Fraser University, CANADA

## Abstract

**Background:**

High-resolution computed tomography has limitations in the assessment of airway wall layers and related remodeling in obstructive lung diseases. Near infrared-based optical coherence tomography (OCT) is a novel imaging technique that combined with bronchoscopy generates highly detailed images of the airway wall. The aim of this study is to identify and quantify human airway wall layers both *ex-vivo* and *in-vivo* by OCT and correlate these to histology.

**Methods:**

Patients with lung cancer, prior to lobectomy, underwent bronchoscopy including *in-vivo* OCT imaging. *Ex-vivo* OCT imaging was performed in the resected lung lobe after needle insertion for matching with histology. Airway wall layer perimeters and their corresponding areas were assessed by two independent observers. Airway wall layer areas (total wall area, mucosal layer area and submucosal muscular layer area) were calculated.

**Results:**

13 airways of 5 patients were imaged by OCT. Histology was matched with 51 *ex-vivo* OCT images and 39 *in-vivo* OCT images. A significant correlation was found between *ex-vivo* OCT imaging and histology, *in-vivo* OCT imaging and histology and *ex-vivo* OCT imaging and *in-vivo* OCT imaging for all measurements (p < 0.0001 all comparisons). A minimal bias was seen in Bland-Altman analysis. High inter-observer reproducibility with intra-class correlation coefficients all above 0.90 were detected.

**Conclusions:**

OCT is an accurate and reproducible imaging technique for identification and quantification of airway wall layers and can be considered as a promising minimal-invasive imaging technique to identify and quantify airway remodeling in obstructive lung diseases.

## Introduction

Airway remodeling is defined by structural changes and thickening of the airway wall, which is seen in several pulmonary diseases, such as asthma and chronic obstructive pulmonary disease (COPD) [1–3]. The identification and severity of airway remodeling is important as it relates to disease severity[4]. Currently, airway remodeling can be assessed by high resolution computed tomography (HRCT)-scan of the chest. However this imaging technique requires patient exposition to ionizing radiation and has limited resolution that hampers visualization and quantification of the different airway wall layers. Bronchial mucosal biopsies taken during bronchoscopy, can visualize the different airway wall layers very precisely but are invasive. Furthermore these biopsies, provide only information of a small selected site of the airways and the processing of biopsies is time consuming and often causes artefacts [5].

Optical coherence tomography (OCT) is a promising real-time high-resolution imaging technique to assess airway remodeling[6, 7]. Using near-infrared light cross-sectional images are created by the backscattering of light by the tissue[8]. For example, in ophthalmology, OCT is used in clinical practice for retina assessment [9] and in cardiology for stent positioning during percutaneous coronary interventions [10]. Former studies have shown that OCT is able to visualize the different airway wall layers including mucosa (epithelium and lamina propria), submucosa (including airway smooth muscle, glands) and cartilage [11–15]. Only limited data are available on the quantification of total airway wall area and the correlation with histology [5, 16] and CT[5, 6]. The feasibility of OCT to quantify separate airway wall layers, including the mucosa and submucosa, and the correlation with histology in human airways in unknown. Furthermore correlating *ex-vivo* and *in-vivo* OCT images has never been done before. The aim of this study is to identify and quantify airway wall layers in *ex-vivo* and *in-vivo* OCT images and correlate these to histology, and assess the inter-observer reproducibility. We hypothesize that: 1) airway wall layer areas assessed on *ex-vivo* OCT images correlate well with matched histology sections, 2) airway wall layer areas assessed on *in-vivo* OCT images correlate well with both *ex-vivo* OCT images and histology sections, and, 3) there is a good inter-observer reproducibility for manually traced OCT airway wall layer perimeters and their corresponding areas.

## Methods

### Study design

**Fig 1:**
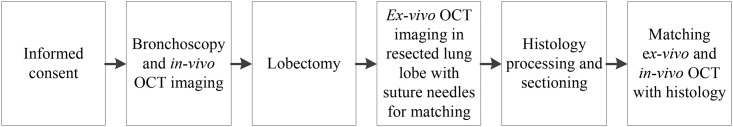
Flow chart of study conduct.

This is a prospective observational cohort study, performed in the Academic Medical Center (AMC) in Amsterdam, the Netherlands. Ethical approval was obtained from the Medical Ethics Committee of the AMC (NL51605.018.14). Fig 1 shows the flow of study conduct.

### Study subjects

Patients with a strong suspicion or tissue proven peripheral non-small cell lung cancer (NSCLC) staged cT1-3N0-1M0 based on a positron emission tomography-computed tomography (PET-CT) and in need of a standard diagnostic bronchoscopic work-up and lobectomy were eligible for the study. Signed informed consent was obtained prior to the procedure.

### In-vivo OCT imaging

OCT images of *in-vivo* airways were acquired using a C7-XR St. Jude Medical Inc. system interfaced with a C7 Dragonfly catheter (Ø 0.9 mm diameter) (St. Jude Medical Inc., St. Paul, MN, USA). After standard diagnostic bronchoscopy, the OCT catheter was inserted through a guide sheath into the working channel of the bronchoscope into the airways of interest where an automated pullback of 5.4 cm was performed (S1A Fig). All airways in the lobe candidate for surgical resection were imaged from subsegmental to segmental airways. Each pullback was repeated at least two times.

### Ex-vivo OCT imaging

Following surgical resection, the lobectomy specimen was subjected to OCT imaging within three hours after removal. In preparation for *ex-vivo* OCT imaging the airways were partially exposed and instilled with phosphate buffered saline. In order to correlate the *ex-vivo* OCT imaging with histology, two to four curved suture needles were inserted in the *in-vivo* OCT imaged airways (S1B Fig). These needles were clearly visible on *ex-vivo* OCT imaging (S1C Fig) and guided matching of *ex-vivo* OCT images with histology sections. *Ex-vivo* OCT imaging was performed similarly to *in-vivo* OCT imaging as described above.

### Histological preparation

**Fig 2:**
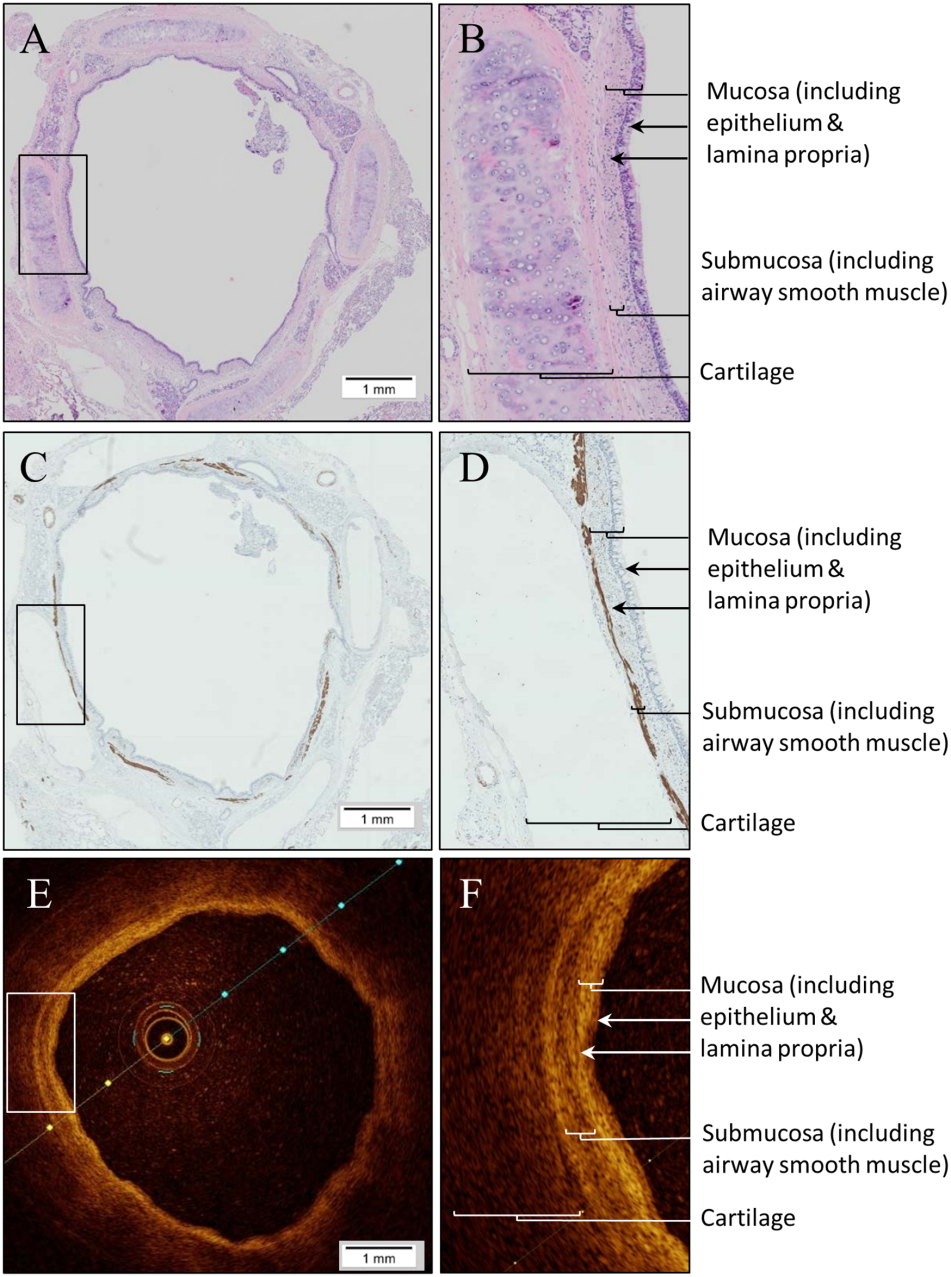
E*x-vivo* OCT cross-sectional image visualizing the different layers of the human airway wall and corresponding histology image. (A) Histology cross section, stained with H&E. (B) Higher magnification view of the square of histology image A, visualizing the different layers of the airway wall of the segmental LLL. (C) Histology cross section, stained with desmin. (D) Higher magnification view of the square of histology image C, visualizing the submucosal muscular layer of the airway wall. (E) Corresponding cross section of OCT of *ex-vivo* airway to histology airway image A and C. (F) Higher magnification view of the square of OCT image E, visualizing the corresponding layers of the airway wall.

After performing *ex-vivo* OCT imaging of the airways, the lobectomy sample was fixed in phosphate buffered formalin overnight. Measured airways were dissected and sectioned according to the sutures needles. Subsequently the tissue samples were dehydrated with increasing concentrations of ethanol for ~4 hours, cleared in xylene and impregnated in paraffin, using a standard tissue processor (A82300001 Excelsior AS Tissue Processor, ThermoFisher Scientific, Waltham, MA, USA). Next, tissues were manually embedded in paraffin. Sections of 4 μm thickness were stained with hematoxylin and eosin (H&E) to visualize the airway wall structures (Fig 2A and 2B). Immunostaining with desmin was used to identify the airway smooth muscle layer (Fig 2C and 2D). We used Philips Digital Pathology Solution 2.3.1.1 to digitalize the histology slides (Philips Electronics, the Netherlands).

### OCT measurements protocol and training

Before analyzing the *ex-vivo* and *in-vivo* OCT images, a protocol was written that defined how to identify and quantify the airway wall layers in OCT images. For training purposes, according to this protocol a test series of OCT images were analyzed by two independent OCT image experts (JH and AG). The test OCT imaging set contained 51 randomly selected OCT images which were obtained from another study. Luminal perimeter (P_L_), outer perimeter of the mucosa (P_muc_) and outer perimeter of the submucosal muscular layer (P_submusc_) were traced.

### OCT—Histology matching

*Ex-vivo* OCT images and histology sections were matched by one observer (JH) based on the needles which were clearly visible in OCT. *In-vivo* OCT images were linked with *ex-vivo* OCT images and histology by matching luminal perimeters and corresponding areas in mm^2^ combined with the distance from reference points and segmentations in corresponding airways.

### OCT and histology measurements

**Fig 3:**
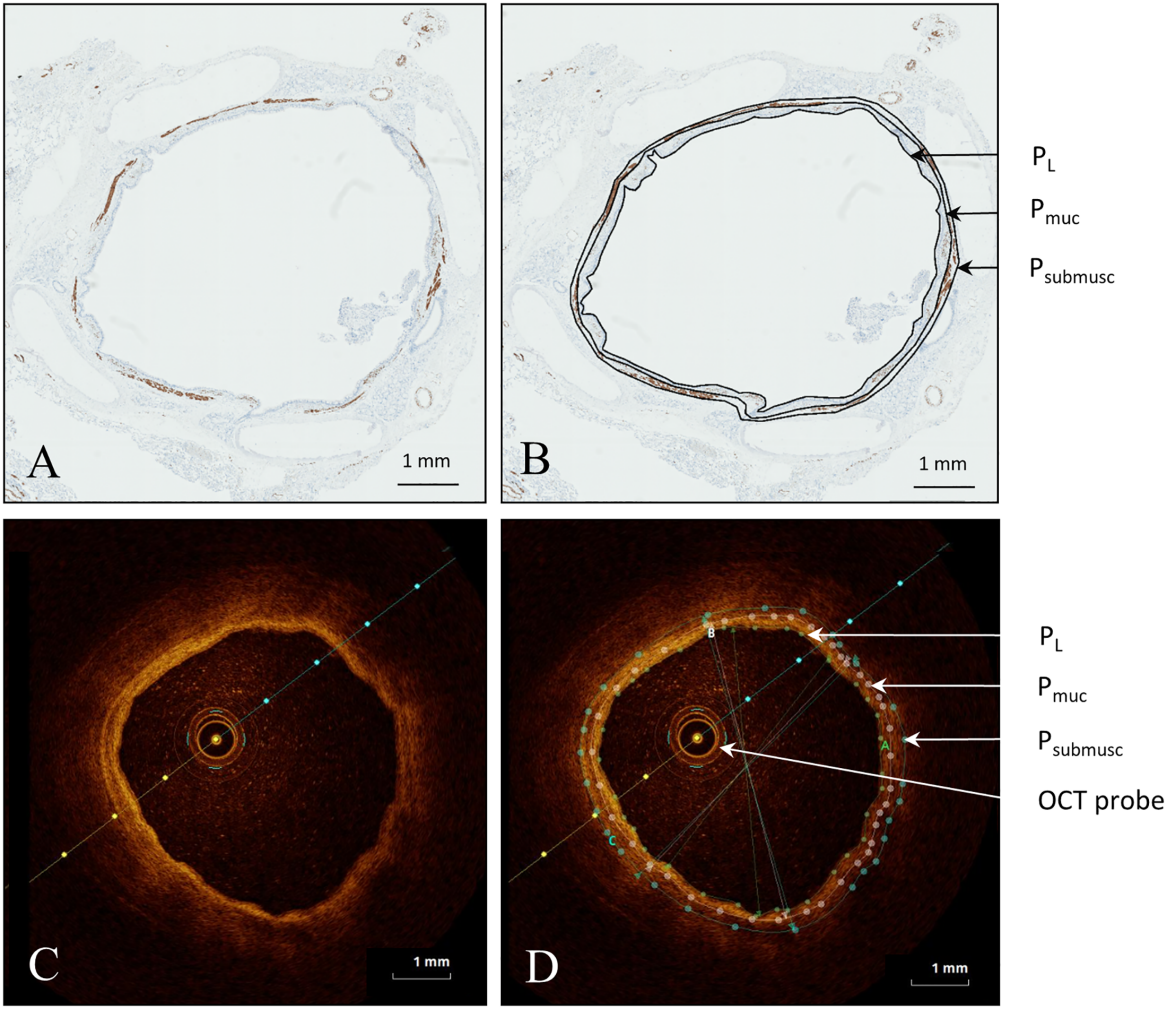
*Ex-vivo* OCT cross-sectional image and corresponding histology image of human airway. (A) Clean histology cross section of human airway of the segmental LLL, stained with desmin. (B) cross section images of histology, stained with desmin, with manually traced perimeters; P_L_: lumen perimeter, P_muc_: mucosal perimeter, P_submusc_: submucosal muscular perimeter. (C) Corresponding cross section of OCT of *ex-vivo* airway to histology airway image A. (D) cross section images of OCT and with manually traced perimeters; P_L_: lumen perimeter, P_muc_: mucosal perimeter, P_submusc_: submucosal muscular perimeter and OCT probe in situ.

We used ImageJ software for Windows (National Institutes of Health, Bethesda, MD, USA) to manually trace the luminal perimeter (P_L_), mucosal perimeter (P_muc_) and submucosal muscular perimeter (P_submusc_) in the desmin stained histology images of our study population (Fig 3A and 3B). The same perimeters were traced in *in-vivo* and *ex-vivo* OCT images using St. Jude Medical Inc. software (Fig 3C and 3D). The criteria for tracing the airway wall layer perimeters in OCT images were based on the differences in light intensity as shown in S2 Fig. From the inside of the airway lumen to the outer wall, the first thin low intensity layer identified is the epithelial layer. The second, high intensity, layer matches the lamina propria layer (the mucosal layer includes the epithelial layer and lamina propria layer). The third, lower intensity, layer is the submucosa layer which includes the airway smooth muscle. The next, very low intensity, layer is the cartilage layer identified by a surrounded thin high intensity layer, the perichondrium.

Subsequently, the luminal areas A_L_ (in mm^2^), mucosal areas A_muc_ (in mm^2^) and submucosal muscular areas A_submusc_ (in mm^2^) corresponding to the traced perimeters P_L_, P_muc_, P_submusc_ were automatically calculated. These areas were used to calculate the surface areas of the different layers; total airway wall area (WA_t_ = A_submusc_−A_L_), mucosal wall layer area (WA_muc_ = A_muc_−A_L_) and submucosal muscular wall layer area (WA_submusc_ = A_submusc_−A_muc_) in mm^2^. Both JH and AG analysed the blinded histology, OCT *ex-vivo* and OCT *in-vivo* images independently in order to assess the inter-observer reproducibility.

### Primary endpoint

The primary endpoint was the correlation between *ex-vivo* OCT and histology for the above described parameters (A_L_, A_muc_, A_submusc_, WA_t_, WA_muc_ and WA_submusc_ in mm^2^).

### Secondary endpoints

Secondary endpoints were the correlation between *in-vivo* OCT and *ex-vivo* OCT and between *in-vivo* OCT and histology for the above described parameters (A_L_, A_muc_, A_submusc_, WA_t_, WA_muc and_ WA_submusc_ and in mm^2^). Furthermore the inter-observer reproducibility of the manually traced perimeters and corresponding areas between two observers was analyzed (A_L_, A_muc_, A_submusc_ in mm^2^).

### Statistical analysis

Data were tested for normality using a D’Agostino and Pearson omnibus normality test and histograms. The relationship between histology and OCT images (*ex-vivo* and *in-vivo*) and the inter-observer reproducibility was determined by using a Pearson correlation coefficient (r) for normally distributed data and the Spearman’s rank correlation coefficient (r) for non-normally distributed data with its least squares linear regression models. The agreement between measurements is shown in Bland-Altman plots. Both analysis were performed in GraphPad Prism version 5.01 (GraphPad Software Inc, San Diego, CA, USA). To analyze if both observers indeed measured the same values we calculated the intra-class correlation coefficient (ICC) using SPSS statistics for Windows version 23.0. The used P values in our analysis were all two sided and sat at a level of statistical significance of P < 0.05.

## Results

From April 2015 until November 2015 5 patients with NSCLC who underwent an lobectomy were included in this study. Patient characteristics are shown in Table 1.

**Table 1:** Patient characteristics undergoing OCT and lobectomy.

	Patient 1	Patient 2	Patient 3	Patient 4	Patient 5
Age (years)	57	69	71	60	64
Sex	Male	Male	Female	Male	Male
FEV_1_% predicted	97	94	90	36	48
COPD GOLD status	n.a.	n.a.	n.a.	GOLD III	GOLD III
Resected lung lobe	RUL	LLL	LLL	LLL	RUL

FEV_1_: forced expiratory volume in one second. COPD: chronic obstructive pulmonary disease. n.a.: not applicable. RUL: right upper lobe. LLL: left lower lobe

### Primary endpoint

**Fig 4:**
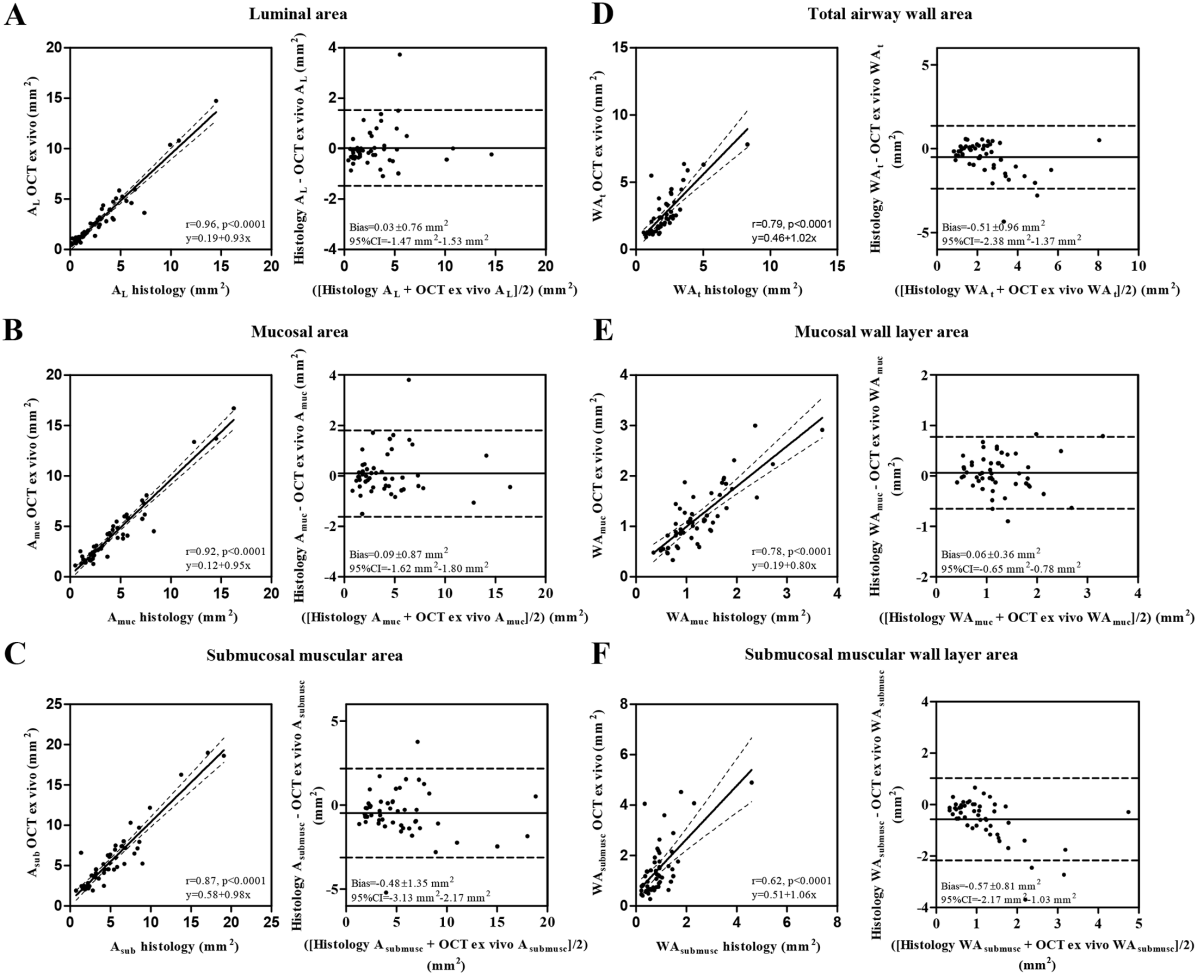
Linear regression analysis and Bland-Altman plots for histology and OCT *ex-vivo* airway wall area measurements (n = 51). (A) A_L_ lumen area in mm^2^. (B) A_muc_ mucosal area in mm^2^. (C) A_submusc_ submucosal muscular area in mm^2^. (D) WA_t_ total airway wall area in mm^2^. (E) WA_muc_ mucosal wall area in mm^2^. (F) WA_submusc_ submucosal muscular wall area in mm^2^.

In total 13 *ex-vivo* airways in 5 patients were imaged with OCT, resulting in 51 matching cross sectional OCT images and histology sections. Airway wall layers could be identified as shown in Figs 2 and 3. Linear regression analysis showed a significant correlation between *ex-vivo* OCT imaging and histology for all parameters (A_L_ r = 0.96, p<0.0001, A_muc_ r = 0.92, p<0.0001, A_sub_ r = 0.87, p<0.0001, WA_t_ r = 0.79, p<0.0001, WA_muc_ r = 0.78, p<0.0001 and WA_submusc_ r = 0.62, p = 0.0001) (Table 2, Fig 4A–4F left graphs). Bland-Altman analysis showed a minimal bias for (all measurements of) these parameters (Fig 4A–4F right graphs). A proportional error was found for mucosal wall area (WA_muc_ in mm^2^) and submucosal muscular wall area (WA_submusc_ in mm^2^).

**Table 2:** Correlation between *ex-vivo* OCT and histology for airway wall area measurements.

Parameter	r	P-value
A_L_	0.96	<0.0001
A_muc_	0.92	<0.0001
A_submusc_	0.87	<0.0001
WA_t_	0.79	<0.0001
WA_muc_	0.78	<0.0001
WA_submusc_	0.62	<0.0001

A_L:_ luminal area. A_muc_: mucosal area. A_submusc:_ submucosal muscular area, WA_t_: total airway wall area. WA_muc_: mucosal wall layer area. WA_submusc_: submucosal muscular wall layer area

### Secondary endpoints

#### Ex-vivo OCT and in-vivo OCT images

A total of 39 *in-vivo* OCT cross sectional images could be compared with their corresponding ex-vivo OCT images. Images were matched according to luminal perimeters for the same airways. High correspondence between *in-vivo* and *ex-vivo* OCT imaging for the luminal area was shown (A_L_ r = 0.99, p<0.0001). Linear regression analysis showed a significant correlation for all other parameters (A_muc_ r = 0.97, p<0.0001, A_submusc_ r = 0.88, p<0.0001, WA_t_ r = 0.54, p<0.0001, WA_muc_ r = 0.68, p<0.0001 and WA_submusc_ r = 0.40, p = 0.0001). Bland-Altman analysis showed a negligible bias between OCT *ex-vivo* and OCT *in-vivo* images (S3 Fig). Similar results were found when *in-vivo* OCT was compared with histology (S4 Fig).

### Inter-observer reproducibility

All 51 *ex-vivo* OCT images and 39 *in-vivo* OCT images were analyzed independently by the two observers. The intra-class correlation coefficients to assess the accuracy of *ex-vivo* and *in-vivo* OCT measurements by the two observers were high for all parameters (S1 Table). Linear regression analysis of the *ex-vivo* OCT measurements between the two observers showed a significant correlation (r ≥0.98, p< 0.0001 for all measurements) (S5 Fig). Bland-Altman plots showed a minimal bias for all three parameters (A_L_ -0.02 (95% CI = -0.22–0.19), A_muc_ -0.06 (95% CI = -0.52–0.41) and A_submusc_ 0.02 (95% CI = -0.99–1.04)(S5 Fig). Comparable results were found for the *in-vivo* OCT measurements between the two observers (S6 Fig).

## Discussion

To the best of our knowledge, this is the first study to show the feasibility of OCT to identify and subsequently quantify separate human airway wall layers showing a strong and significant correlation with histology for both *ex-vivo* and *in-vivo* OCT images. Besides, this is the first report comparing *ex-vivo* and *in-vivo* OCT imaging and showing its significant correlation. Importantly, a high inter-observer reproducibility was detected between two independent observers.

The correlations for separate airway wall areas measured by OCT and histology were less strong when compared to the previous animal study performed in porcine lungs, which are known to have more widespread airway cartilage than human airways [16]. This makes it easier to distinguish the different perimeters in OCT images of porcine airways[17]. The proportional error in Bland-Altman analysis of WA_submusc_ (mm^2^) and WA_t_ (mm^2^) for *ex-vivo* OCT imaging versus histology, suggests that a small systematic difference between histology and OCT, is present. Probably, this is the result of formalin fixation, alcohol dehydration and paraffin embedding, which are known to cause tissue shrinkage, however without disturbing the intrinsic proportions of the various tissue layers. The high correlation we found for the lumen and total airway wall quantification is comparable to the *in-vivo* OCT study performed by Chen et al[5].

Since we could not use suture needles as landmarks for matching the OCT *in-vivo* images with histology, we used a corresponding inner luminal area from the same airway instead. A decrease of the airway lumen after resection and especially after histological processing can be expected. This can contribute to the observed differences between the *in-vivo* OCT and *ex-vivo* OCT measurements and *in-vivo* OCT and histological measurements. However, the correlation for A_muc_ and A_submusc_ remained strong and significant for *in-vivo* OCT imaging compared to histology and suggests that an exact match is not necessary when you measure the same airway with a corresponding lumen. A previous study assessing insertion-reinsertion reproducibility of the total airway wall area in OCT found similar results and stated that heterogeneity in airway wall structure seemed to be relatively small[18].

This study has multiple strengths. First, all measurements were independently assessed by two observers, making it possible to analyze the inter-observer reproducibility. By using an anonymized test series of different patients, we avoided creating bias by already seeing the OCT images from our own cohort. Based on histology and the OCT test series both observers with knowledge of the histology of airway walls were able to analyze OCT images of the airway wall. With high intra-class correlation coefficients and a negligible bias for all measurements between two independent observers this study shows that OCT imaging has a strong inter-observer reproducibility. The strong inter-observer reproducibility in both *ex-vivo* and *in-vivo* OCT imaging, confirms data from previous studies[16, 18]. Second, a large sample size of 51 matched cross sectional OCT and histology images were analyzed. Third, the unique study method, where human airways were measured with OCT both *in-vivo* and *ex-vivo* and subsequently compared with each other and with histology, ensured a reliable comparison. Finally, we believe that the method of airway wall layer quantification as executed in this study is of interest. Since subtracting airway wall area measurements from one another results in areas of the airway wall layer that are independent of the shape of the airway.

There are several limitations to this study. First, not all *ex-vivo* airways were also measured with OCT *in-vivo*, therefore 39 of the 52 *ex-vivo* OCT images had a corresponding *in-vivo* OCT image. Since it was not possible to use sutures during OCT *in-vivo* imaging, the luminal area and distance to reference points and segmentations was used to match with histology. In addition, matching was done by a single person. Second, our cohort contained a heterogeneous population with both subjects with a non-obstructive lung function and subjects with COPD. However, in these 5 patients several airways were imaged, creating 51 histology—OCT *ex-vivo* matches. Since the aim of this study was to correlate histology to OCT images independent of the health status of the airway wall, the heterogeneity of the study population does not interfere with the study aim. Another possible limitation is generated by artefacts after processing histology tissue. A well-known artefact is shrinkage of histology tissue caused by fixation[19]. This could potentially have caused the small proportional error seen in the Bland-Altman analysis. In addition, OCT imaging artifacts can contribute to the found differences between OCT and histological measurements. As shown in Figs 2 and 3 the sensitivity decreases with the distance between the probe and the airway wall. Furthermore minimal artefacts are expected from the angle of the airway wall surface relative to the light beam, the refractive index radial calibration and refractive effects.

One major advantage of OCT over histology is that OCT is able to measure airway segments real-time in their natural state *in-vivo*. With OCT being significantly correlated to histology and easy to learn with high reproducibility, it could be an ideal instrument to assess airway wall remodeling, understand airway disease pathogenesis and eventually monitor and evaluate treatment results over time in patients with airway diseases such as asthma[20]. For instance, there is evidence that Bronchial Thermoplasty (BT) induces a reduction in airway smooth muscle (ASM) mass in severe asthma[21]. As OCT is able to visualize and quantify the different airway wall layers in a specific airway segment, it could potentially detect changes in the submucosal muscular layer, containing the ASM after BT. As such OCT may serve as an ideal BT treatment evaluation instrument and might be used for identification of patients that have a large ASM mass. In the future, combining OCT with technical advancements such as polarisation will make it possible to visualize and quantify the ASM itself, which could be of added value in these patients [22]. For this purpose, although the high inter-observer reproducibility of OCT measurements are reassured, automated software for airway wall measurements is highly needed.

In conclusion, OCT is an accurate and reproducible imaging technique for identification and quantification of the airway wall areas in total and in sublayers. OCT can be considered a promising non-invasive imaging technique to identify and quantify airway remodeling in patients with obstructive lung diseases.

## Supporting information

S1 Table Inter-observer reproducibility of OCT measurements between two independent observers. (PDF)

S1 Fig Bronchoscopic OCT imaging technique. (A) Bronchoscopic view: *In-vivo* OCT imaging with OCT catheter (left arrow) outside the sheet (right arrow) in the posterior segment (RB9) of the right lower lobe. The medial-basal segment (RB7) is used as reference point for the end of the pullback track of 5.4 cm marked by a metal part (left arrow). (B) Resected lung lobe with 3 suture needle marks (long arrows) through the lumen of the airway. OCT catheter placed in airway with needle marks (short arrow). (C) OCT cross-section of an *ex-vivo* imaged airway with a needle mark visible (long arrow). OCT probe visible in the center of the airway (short arrow).
(TIF)

S2 Fig OCT criteria for identification of airway wall structures and layers. (A) OCT image of the airway wall of a segmental airway of the left lower lobe. (B) Manual tracing of perimeters based on differences in light intensities of the airway wall layers. From right to left the dotted lines represent; luminal perimeter, epithelial perimeter, mucosal perimeter, submucosal perimeter. (C) Corresponding annotated airway wall layers based on differences in light intensities. From right to left: first, low intensity, layer is the epithelial layer. The second, high intensity, layer matches the lamina propria layer. The third, low intensity, layer the submucosa including the airway smooth muscle. The next, very low intensity, layer is the cartilage layer which is identified by a surrounded thin high intensity layer, the perichondrium.
(TIF)

S3 Fig Linear regression analysis and Bland-Altman plots for OCT *ex-vivo* and OCT *in-vivo* airway wall area measurements (n = 39). (A) A_L_ lumen area in mm^2^. (B) A_muc_ mucosal area in mm^2^. (C) A_submusc_ submucosal muscular area in mm^2^.
(TIF)

S4 Fig Linear regression analysis and Bland-Altman plots for histology and OCT *in-vivo* airway wall area measurements (n = 39). (A) A_L_ lumen area in mm^2^. (B) A_muc_ mucosal area in mm^2^. (C) A_submusc_ submucosal muscular area in mm^2^.
(TIF)

S5 Fig Linear regression analysis and Bland-Altman plots for *ex-vivo* OCT airway wall measurements between two observers (n = 51). (A) A_L_ lumen area in mm^2^. (B) A_muc_ mucosal area in mm^2^. (C) A_submusc_ submucosal muscular area in mm^2^.
(TIF)

S6 Fig Linear regression analysis and Bland-Altman plots for *in-vivo* OCT airway wall measurements between two observers (n = 39). (A) A_L_ lumen area in mm^2^. (B) A_muc_ mucosal area in mm^2^. (C) A_submusc_ submucosal muscular area in mm^2^.
(TIF)

S1 File Supporting information database. (PDF)

## Abbreviations

AMC: academic Medical Center
ASM: airway smooth muscle
BT: bronchial thermoplasty
COPD: chronic pulmonary obstructive disease
H&E: hematoxylin and eosin
HRCT: high resolution computed tomography
ICC: intra-class correlation
NSCLC: non-small cell lung cancer
OCT: optical coherence tomography
PET-CT: positron emission tomography-computed tomography
