# Supplementary material for: Optical coherence tomography for identification and quantification of human airway wall layers
Source: PLoS One. 2017 Oct 5;12(10):e0184145. doi: 10.1371/journal.pone.0184145 (PMC5628810; doi:10.1371/journal.pone.0184145)
Supplement: S1 Table — (PDF) [file pone.0184145.s001.pdf]

Table S1

Inter-observer reproducibility of OCT measurements between two independent observers.

	***Ex-vivo* OCT imaging (n = 51)**			***In-vivo* OCT imaging (n = 39)**		
Parameter	ICC	95% CI	P-value	ICC	95% CI	P-value
A_L (mm^2)	0.999	0.999-1.000	<0.0001	0.999	0.999-1.000	<0.0001
A_{muc} (mm^2)	0.997	0.995-0.998	<0.0001	0.997	0.993-0.999	<0.0001
$A_{submusc}$ (mm^2)	0.991	0.985-0.995	<0.0001	0.983	0.959-0.992	<0.0001

ICC: intraclass correlation. $A_{L:}$ luminal area. A_{muc}: mucosal area. $A_{submusc:}$ submucosal muscular area.
