# Supplementary figures and images for: Optical coherence tomography for identification and quantification of human airway wall layers
Source: PLoS One. 2017 Oct 5;12(10):e0184145. doi: 10.1371/journal.pone.0184145 (PMC5628810; doi:10.1371/journal.pone.0184145)

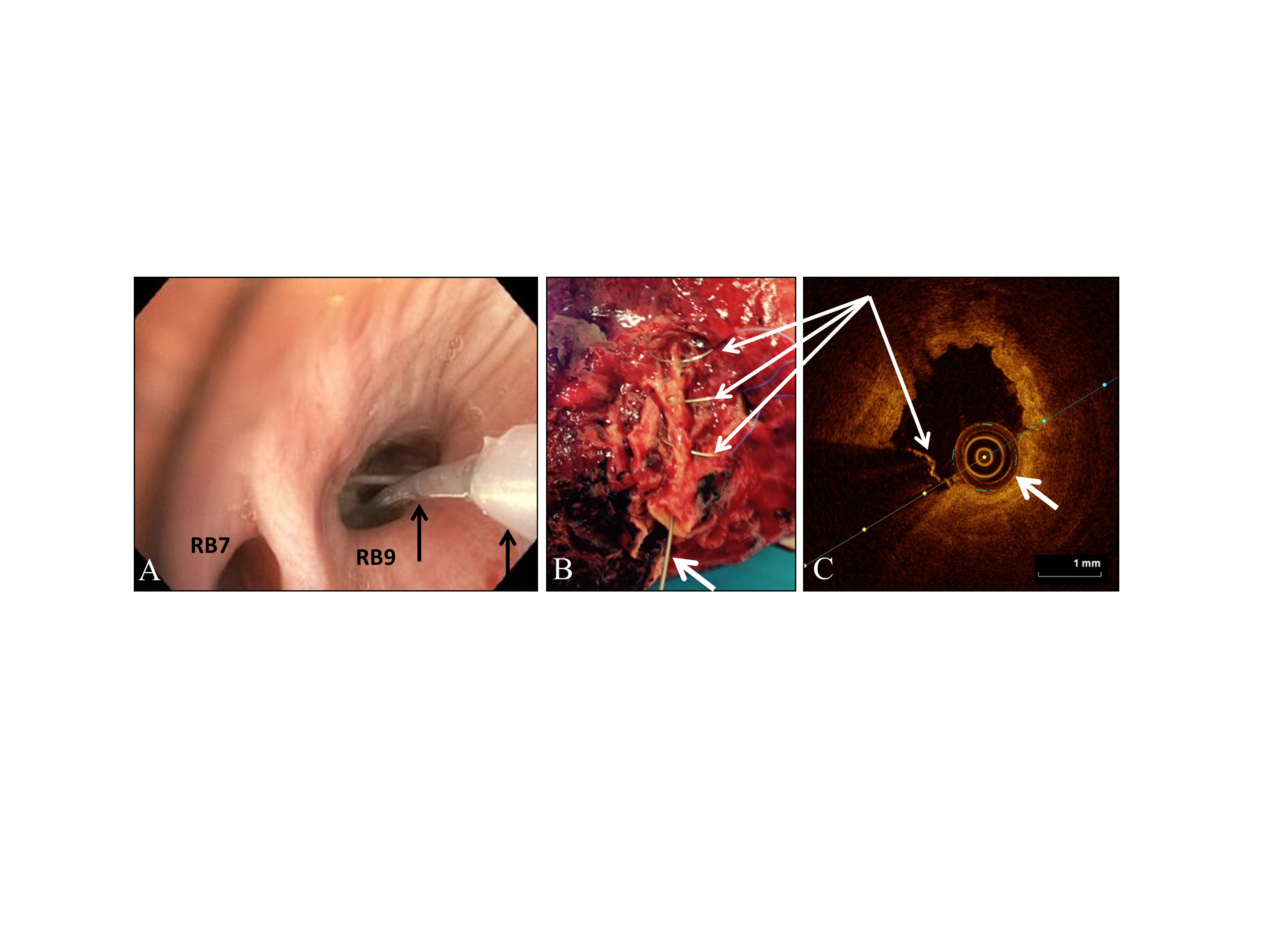

Supplement: S1 Fig — (A) Bronchoscopic view: In-vivo OCT imaging with OCT catheter (left arrow) outside the sheet (right arrow) in the posterior segment (RB9) of the right lower lobe. The medial-basal segment (RB7) is used as reference point for the end of the pullback track of 5.4 cm marked by a metal part (left arrow). (B) Resected lung lobe with 3 suture needle marks (long arrows) through the lumen of the airway. OCT catheter placed in airway with needle marks (short arrow). (C) OCT cross-section of an ex-vivo imaged airway with a needle mark visible (long arrow). OCT probe visible in the center of the airway (short arrow). (TIF) [file pone.0184145.s002.tif]

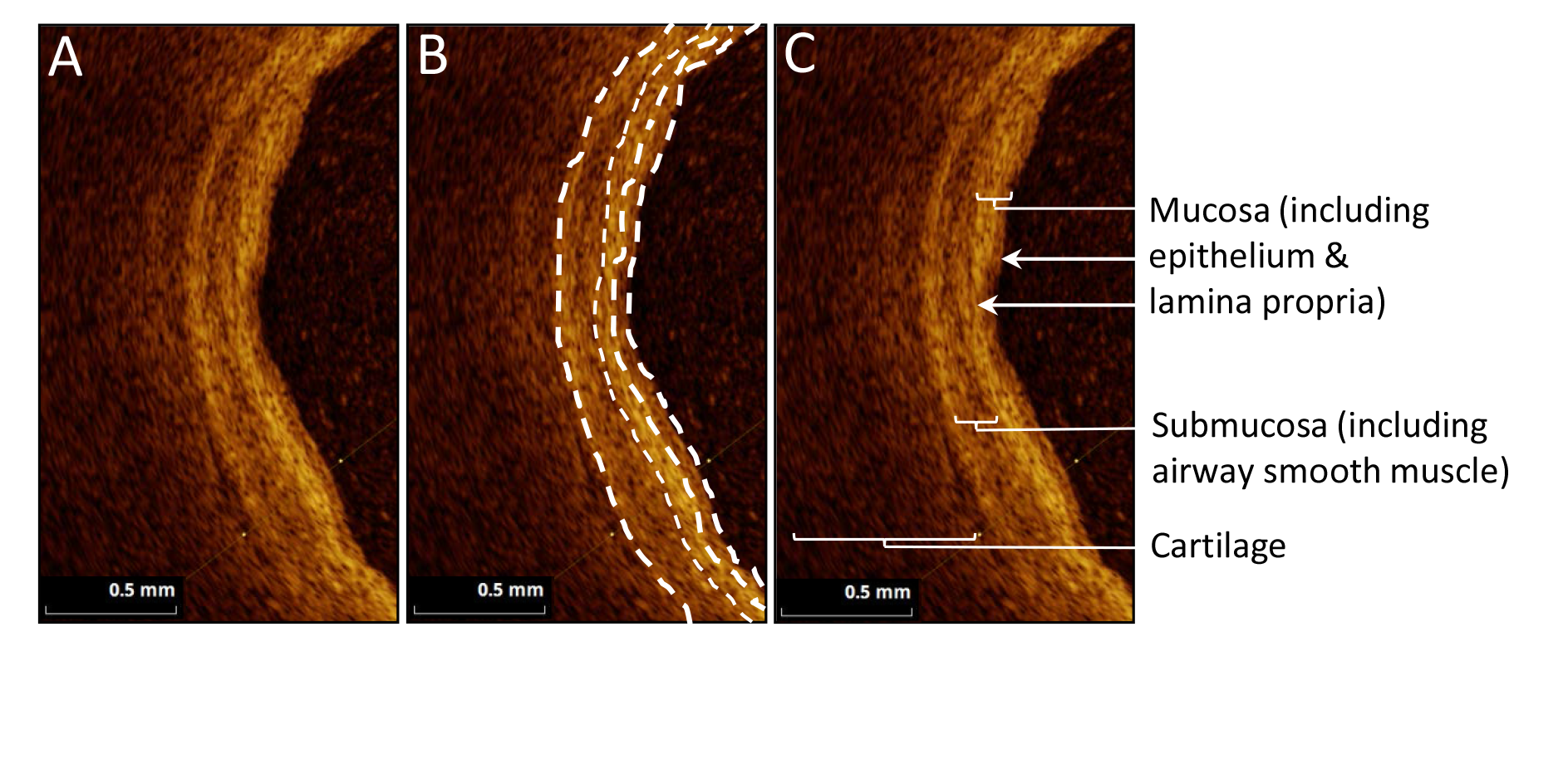

Supplement: S2 Fig — (A) OCT image of the airway wall of a segmental airway of the left lower lobe. (B) Manual tracing of perimeters based on differences in light intensities of the airway wall layers. From right to left the dotted lines represent; luminal perimeter, epithelial perimeter, mucosal perimeter, submucosal perimeter. (C) Corresponding annotated airway wall layers based on differences in light intensities. From right to left: first, low intensity, layer is the epithelial layer. The second, high intensity, layer matches the lamina propria layer. The third, low intensity, layer the submucosa including the airway smooth muscle. The next, very low intensity, layer is the cartilage layer which is identified by a surrounded thin high intensity layer, the perichondrium. (TIF) [file pone.0184145.s003.tif]

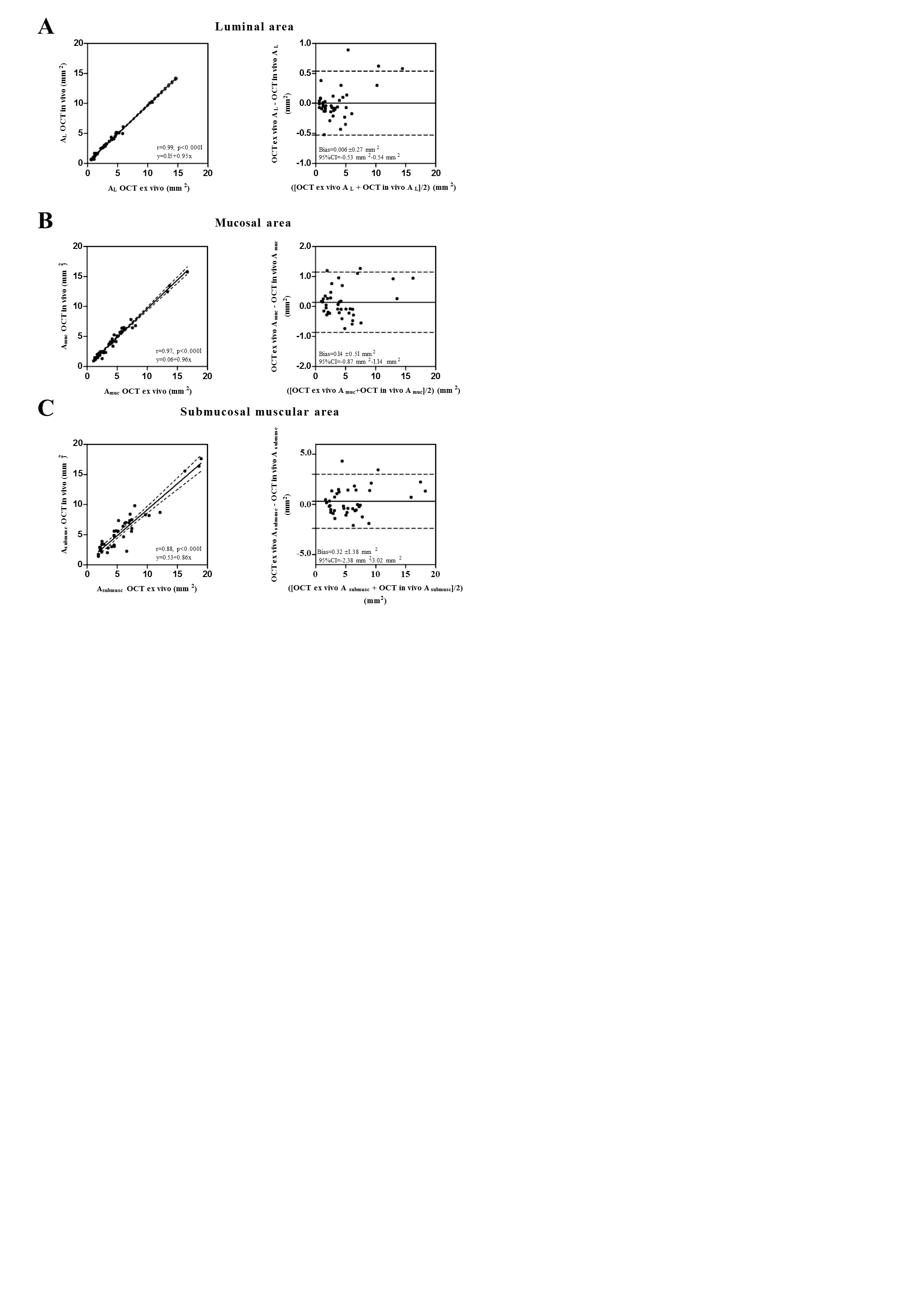

Supplement: S3 Fig — (A) AL lumen area in mm2. (B) Amuc mucosal area in mm2. (C) Asubmusc submucosal muscular area in mm2. (TIF) [file pone.0184145.s004.tif]

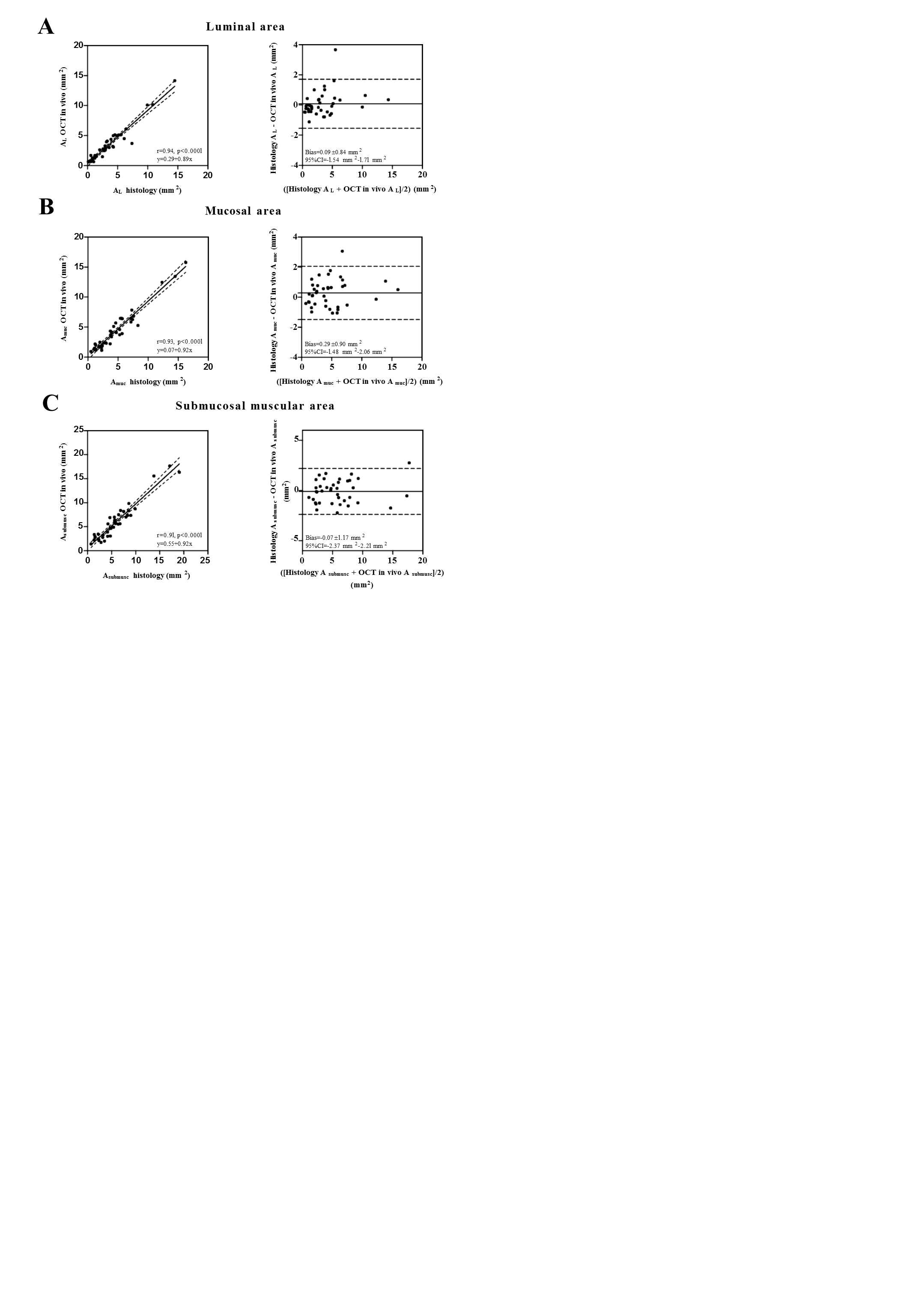

Supplement: S4 Fig — (A) AL lumen area in mm2. (B) Amuc mucosal area in mm2. (C) Asubmusc submucosal muscular area in mm2. (TIF) [file pone.0184145.s005.tif]

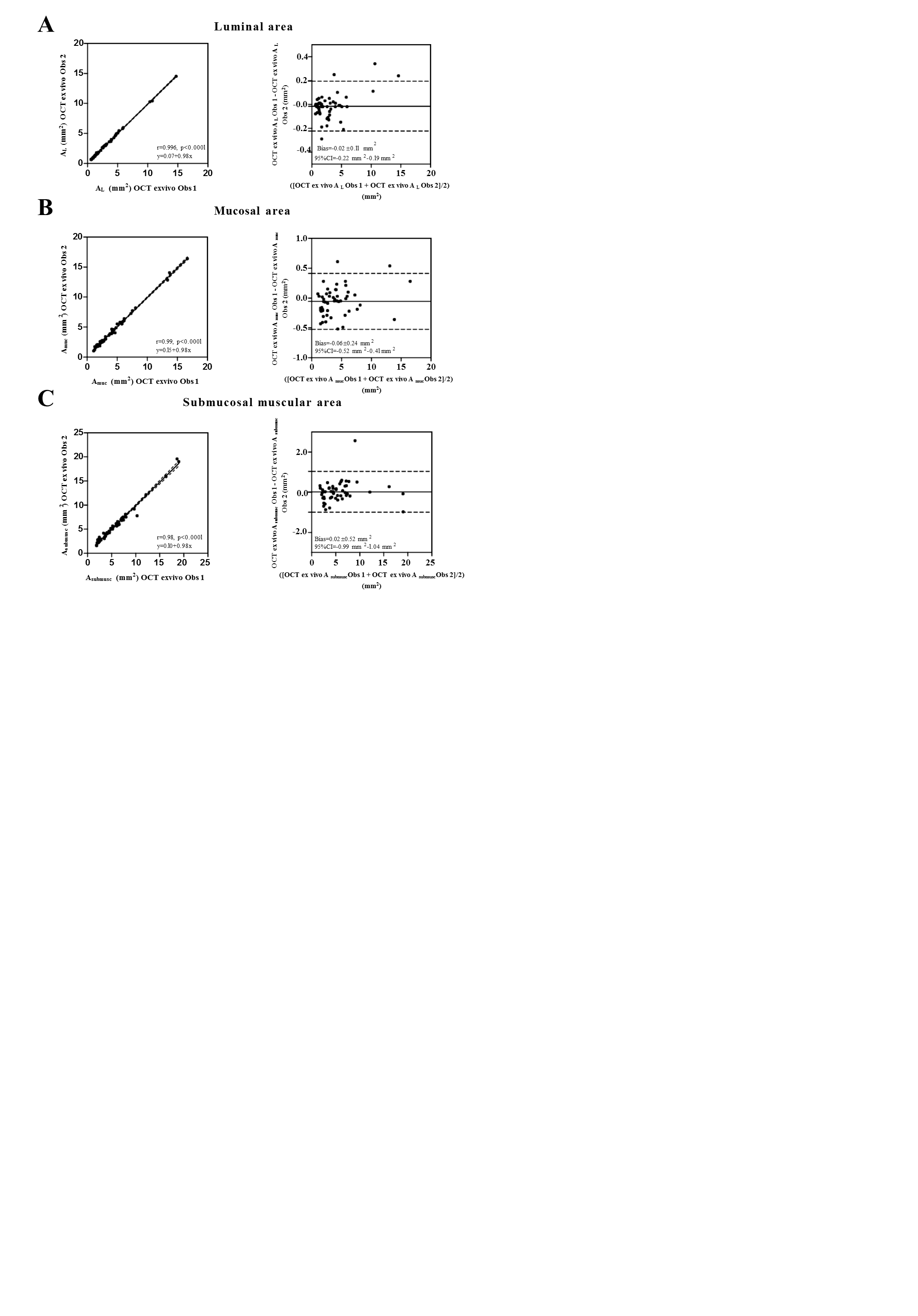

Supplement: S5 Fig — (A) AL lumen area in mm2. (B) Amuc mucosal area in mm2. (C) Asubmusc submucosal muscular area in mm2. (TIF) [file pone.0184145.s006.tif]

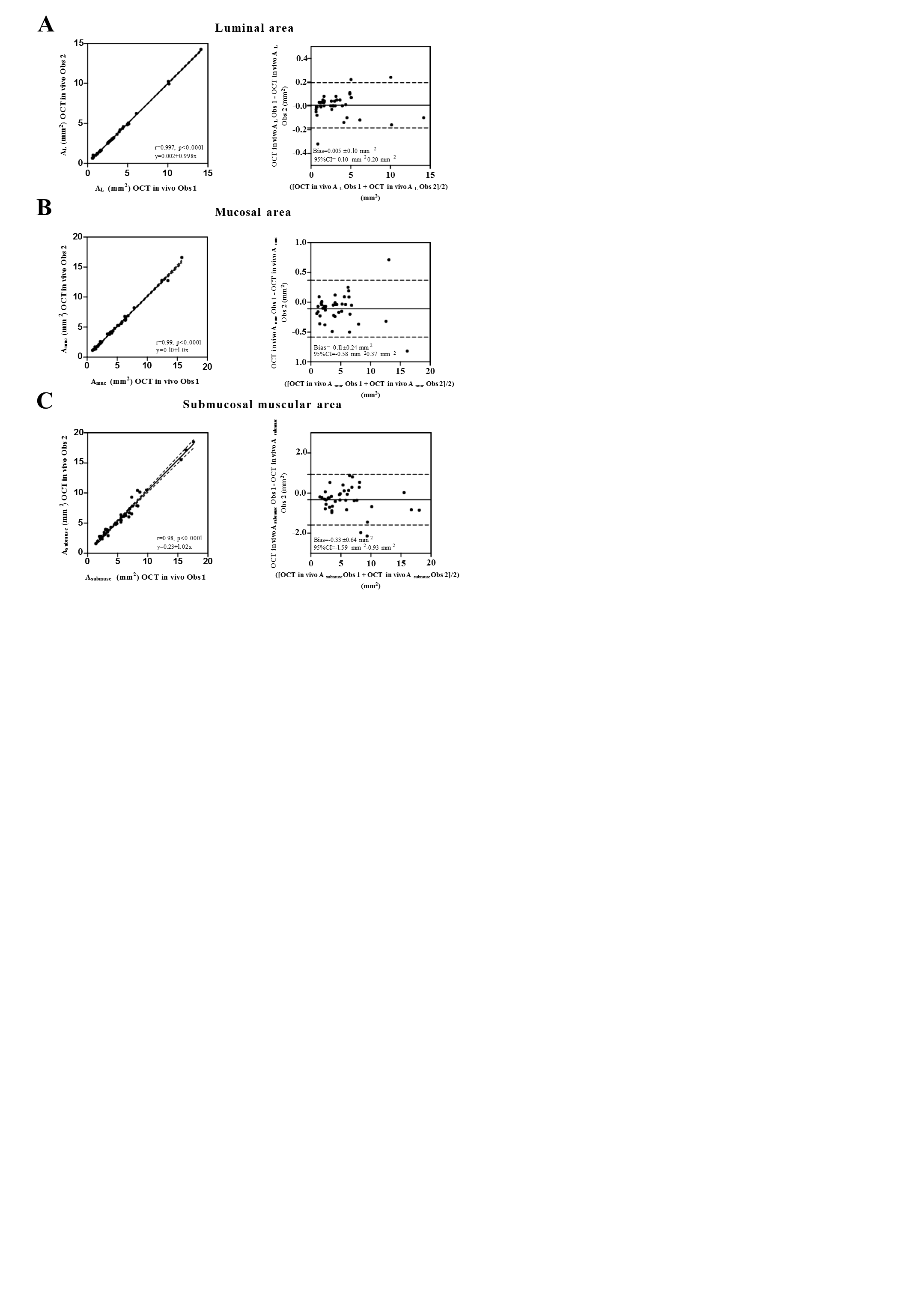

Supplement: S6 Fig — (A) AL lumen area in mm2. (B) Amuc mucosal area in mm2. (C) Asubmusc submucosal muscular area in mm2. (TIF) [file pone.0184145.s007.tif]
