# Supplementary material for: Optical coherence tomography for identification and quantification of human airway wall layers
Source: PLoS One. 2017 Oct 5;12(10):e0184145. doi: 10.1371/journal.pone.0184145 (PMC5628810; doi:10.1371/journal.pone.0184145)
Supplement: S1 File — (PDF) [file pone.0184145.s008.pdf]

	PL	Pmuc	Psubmusc	WAmuc	WAmuc(%)	Wasubmu	WAsubmuc(	WAt	WAt(%)
A1 histology									
IIIA	1,22	2,75	4,20	1,53	55,64	1,45	34,52	2,98	70,95
IIIB	1,45	3,07	4,75	1,62	52,77	1,68	35,37	3,30	69,47
IIIC	0,79	2,26	3,56	1,47	65,04	1,3	36,52	2,77	77,81
IIID	0,59	1,38	2,27	0,79	57,25	0,89	39,21	1,68	74,01
A2 histology									
IIA	4,02	5,76	6,65	1,74	30,21	0,89	13,38	2,63	39,55
IIB	1,79	3,20	4,13	1,41	44,06	0,93	22,52	2,34	56,66
IIIA	10,80	14,50	19,10	3,7	25,52	4,6	24,08	8,30	43,46
IIIB	3,93	5,72	7,51	1,79	31,29	1,79	23,83	3,58	47,67
VB	4,87	7,59	9,90	2,72	35,84	2,31	23,33	5,03	50,81
A3 histology									
IB	3,00	4,71	5,64	1,71	36,31	0,93	16,49	2,64	46,81
IC	5,20	7,15	8,58	1,95	27,27	1,43	16,67	3,38	39,39
IF	1,49	2,63	3,22	1,14	43,35	0,59	18,32	1,73	53,73
IIA	2,74	4,24	4,98	1,5	35,38	0,74	14,86	2,24	44,98
IIB	1,41	2,38	3,19	0,97	40,76	0,81	25,39	1,78	55,80
IIC	2,33	4,00	4,94	1,67	41,75	0,94	19,03	2,61	52,83
IID	1,26	2,15	3,09	0,89	41,40	0,94	30,42	1,83	59,22
IIE1	1,66	2,45	2,82	0,79	32,24	0,37	13,12	1,16	41,13
IIE2	1,35	2,24	2,68	0,89	39,73	0,44	16,42	1,33	49,63
IIG	1,31	2,04	2,31	0,73	35,78	0,27	11,69	1,00	43,29
A4 histology									
IB	2,96	3,93	4,68	0,97	24,68	0,75	16,03	1,72	36,75
ID	0,23	1,02	1,37	0,79	77,45	0,35	25,55	1,14	83,21
IE	1,05	2,31	2,82	1,26	54,55	0,51	18,09	1,77	62,77
IG	0,16	0,51	0,74	0,35	68,63	0,23	31,08	0,58	78,38
IIIA	9,95	12,32	13,80	2,37	19,24	1,48	10,72	3,85	27,90
IIIB	14,50	16,25	17,10	1,75	10,77	0,85	4,97	2,60	15,20
IIID	0,90	2,39	3,18	1,49	62,34	0,79	24,84	2,28	71,70
IIIF	0,81	1,91	2,31	1,1	57,59	0,4	17,32	1,50	64,94
IIIG	0,49	1,11	1,57	0,62	55,86	0,46	29,30	1,08	68,79
VA	2,02	3,93	4,86	1,91	48,60	0,93	19,14	2,84	58,44
VB	2,85	5,25	6,38	2,4	45,71	1,13	17,71	3,53	55,33
VC	0,58	1,21	1,43	0,63	52,07	0,22	15,38	0,85	59,44
VD	0,62	1,46	1,86	0,84	57,53	0,4	21,51	1,24	66,67
VE	0,66	1,47	1,76	0,81	55,10	0,29	16,48	1,10	62,50
VF	0,90	1,53	1,98	0,63	41,18	0,45	22,73	1,08	54,55
A5 histology									
IA	4,28	5,39	6,87	1,11	20,59	1,48	21,54	2,59	37,70
IB	4,55	5,62	6,46	1,07	19,04	0,84	13,00	1,91	29,57
IC	3,28	4,63	5,57	1,35	29,16	0,94	16,88	2,29	41,11
ID	3,16	4,29	5,68	1,13	26,34	1,39	24,47	2,52	44,37
IE	0,82	1,31	1,58	0,49	37,40	0,27	17,09	0,76	48,10
IF	1,19	1,83	2,39	0,64	34,97	0,56	23,43	1,20	50,21
IIA	6,44	7,31	8,60	0,87	11,90	1,29	15,00	2,16	25,12
IIB	7,37	8,31	8,99	0,94	11,31	0,68	7,56	1,62	18,02
IID	4,21	5,25	5,85	1,04	19,81	0,6	10,26	1,64	28,03
IVB	5,60	7,40	8,36	1,8	24,32	0,96	11,48	2,76	33,01
IVC	4,32	5,69	6,73	1,37	24,08	1,04	15,45	2,41	35,81
IVD	3,60	4,72	5,44	1,12	23,73	0,72	13,24	1,84	33,82
IVE	2,59	3,80	4,71	1,21	31,84	0,91	19,32	2,12	45,01
VA	6,09	7,17	7,99	1,08	15,06	0,82	10,26	1,90	23,78
VB	2,93	3,72	4,28	0,79	21,24	0,56	13,08	1,35	31,54
VC	2,48	3,70	4,19	1,22	32,97	0,49	11,69	1,71	40,81
VD	1,45	1,99	2,21	0,54	27,14	0,22	9,95	0,76	34,39

	PL	Pmuc	Psubmusc	WAmuc	WAmuc(%)	Wasubmu	WAsubmuc(	WAt	WAt(%)
A1 OCT ex vivo									
Apicaal 2 hechtingen 1 met naald 240	1,57	2,64	4,09	1,07	40,53	1,45	35,45	2,52	61,61
Apicaal 2 hechtingen 1 met naald 137	1,57	2,77	4,53	1,20	43,32	1,76	38,85	2,96	65,34
Apicaal 2 hechtingen 1 met naald 123	0,87	1,8	3,37	0,93	51,67	1,57	46,59	2,50	74,18
Apicaal 2 hechtingen 1 met naald 99	0,60	1,42	2,18	0,82	57,75	0,76	34,86	1,58	72,48
A2 OCT ex vivo									
Ant ant naald 3 504	3,90	5,83	8,02	1,93	33,10	2,19	27,31	4,12	51,37
Ant ant naald 3 464	1,87	3,08	5,18	1,21	39,29	2,1	40,54	3,31	63,90
Ant post 2 naald 1.1 539	10,79	13,7	18,59	2,91	21,24	4,89	26,30	7,80	41,96
Ant post 2 naald 1.1 487	3,95	5,79	10,31	1,84	31,78	4,52	43,84	6,36	61,69
Post ant naald 1 486	5,85	8,08	12,15	2,23	27,60	4,07	33,50	6,30	51,85
A3 OCT ex vivo									
OCT 1.3 naalden 3 398	2,97	4,83	7,46	1,86	38,51	2,63	35,25	4,49	60,19
OCT 1.3 naalden 3 318	5,24	7,55	9,69	2,31	30,60	2,14	22,08	4,45	45,92
OCT 1.3 naalden 3 136	1,51	3,09	4,50	1,58	51,13	1,41	31,33	2,99	66,44
OCT 2 rechts 2 naalden.4 481	2,99	4,65	6,37	1,66	35,70	1,72	27,00	3,38	53,06
OCT 2 rechts 2 naalden.4 421	1,72	2,99	4,06	1,27	42,47	1,07	26,35	2,34	57,64
OCT 2 rechts 2 naalden.4 382	2,50	4,12	5,30	1,62	39,32	1,18	22,26	2,80	52,83
OCT 2 rechts 2 naalden.1 292	1,31	2,16	3,70	0,85	39,35	1,54	41,62	2,39	64,59
OCT 2 rechts 2 naalden.3 252	1,66	2,55	3,73	0,89	34,90	1,18	31,64	2,07	55,50
OCT 2 rechts 2 naalden.3 260	1,47	2,41	3,77	0,94	39,00	1,36	36,07	2,30	61,01
OCT 2 rechts 2 naalden.3 116	1,28	1,61	2,43	0,33	20,50	0,82	33,74	1,15	47,33
A4 OCT ex vivo									
LB8 rechts.4 397	2,46	4,33	6,25	1,87	43,19	1,92	30,72	3,79	60,64
LB8 rechts.4 318	1,09	2,53	6,58	1,44	56,92	4,05	61,55	5,49	83,43
LB8 rechts.4 279	0,67	1,26	1,90	0,59	46,83	0,64	33,68	1,23	64,74
LB8 rechts.4 041	0,62	1,1	1,88	0,48	43,64	0,78	41,49	1,26	67,02
LB9 onder.5 hechting laatste verplaatst 459	10,38	13,38	16,26	3,00	22,42	2,88	17,71	5,88	36,16
LB9 onder.5 hechting laatste verplaatst 416	14,73	16,69	18,96	1,96	11,74	2,27	11,97	4,23	22,31
LB9 onder.5 hechting laatste verplaatst 251	1,22	2,13	3,52	0,91	42,72	1,39	39,49	2,30	65,34
LB9 boven.1 353	0,97	1,74	2,33	0,77	44,25	0,59	25,32	1,36	58,37
LB9 boven.1 213	0,82	1,3	2,26	0,48	36,92	0,96	42,48	1,44	63,72
LB10 boven.2 4 naalden 474	2,58	4,32	6,08	1,74	40,28	1,76	28,95	3,50	57,57
LB10 boven.2 4 naalden 425	2,22	3,79	7,38	1,57	41,42	3,59	48,64	5,16	69,92
LB10 boven.2 4 naalden 319	1,18	2	2,45	0,82	41,00	0,45	18,37	1,27	51,84
LB10 boven.2 4 naalden 297	0,93	1,5	2,04	0,57	38,00	0,54	26,47	1,11	54,41
LB10 boven.2 4 naalden 258	1,03	1,63	2,46	0,60	36,81	0,83	33,74	1,43	58,13
LB10 boven.2 4 naalden 208	0,94	1,4	2,17	0,46	32,86	0,77	35,48	1,23	56,68
A5 OCT ex vivo									
Ant lat met 3 naalden.3 512	4,73	5,97	7,15	1,24	20,77	1,18	16,50	2,42	33,85
Ant lat met 3 naalden.3 465	5,05	6,16	7,41	1,11	18,02	1,25	16,87	2,36	31,85
Ant lat met 3 naalden.3 456	4,37	5,47	6,97	1,10	20,11	1,5	21,52	2,60	37,30
Ant lat met 3 naalden.3 386	4,00	5,01	5,97	1,01	20,16	0,96	16,08	1,97	33,00
Ant lat met 3 naalden.3 352	1,21	1,73	2,13	0,52	30,06	0,4	18,78	0,92	43,19
Ant lat met 3 naalden.3 324	1,21	1,78	2,45	0,57	32,02	0,67	27,35	1,24	50,61
Post rechts met 3 naald.3 477	5,94	7,29	7,92	1,35	18,52	0,63	7,95	1,98	25,00
Post rechts met 3 naald.3 445	3,63	4,51	5,24	0,88	19,51	0,73	13,93	1,61	30,73
Post rechts met 3 naald.1 412	3,11	4,19	4,82	1,08	25,78	0,63	13,07	1,71	35,48
Api lat 2 naalden.1 472	4,80	6,16	7,11	1,36	22,08	0,95	13,36	2,31	32,49
Api lat 2 naalden.1 431	2,95	4,07	5,19	1,12	27,52	1,12	21,58	2,24	43,16
Api lat 2 naalden.1 332	2,79	3,86	4,48	1,07	27,72	0,62	13,84	1,69	37,72
Api lat 2 naalden.1 303	2,68	3,64	4,54	0,96	26,37	0,9	19,82	1,86	40,97
Api mediaal 2 naalden.4 364	4,59	5,74	6,48	1,15	20,03	0,74	11,42	1,89	29,17
Api mediaal 2 naalden.4 314	3,18	4,23	4,50	1,05	24,82	0,27	6,00	1,32	29,33
Api mediaal 2 naalden.4 246	1,34	1,99	2,48	0,65	32,66	0,49	19,76	1,14	45,97
Api mediaal 2 naalden.4 226	1,67	2,22	2,82	0,55	24,77	0,6	21,28	1,15	40,78

	PL	Pmuc	Psubmusc	WAmuc	WAmuc(%)	Wasubmu	WAsubmuc(	WAt	WAt(%)
A1 OCT in vivo									
RB apicaal links tak.1 415	1,66	2,36	3,02	0,70	29,66	0,66	21,85	1,36	45,03
RB apicaal links tak.1 402	1,54	2,3	3,05	0,76	33,04	0,75	24,59	1,51	49,51
RB apicaal links tak.1 283	0,79	1,45	2,01	0,66	45,52	0,56	27,86	1,22	60,70
RB apicaal links tak.1 259	0,67	1,17	2,38	0,50	42,74	1,21	50,84	1,71	71,85
A2 OCT in vivo									
LOK Anterior posterior.6 441	10,17	13,44	16,35	3,27	24,33	2,91	17,80	6,18	37,80
LOK Anterior posterior.6 415	4,38	6,38	8,2	2,00	31,35	1,82	22,20	3,82	46,59
LOK posterior anterior.4 465	4,96	6,81	8,7	1,85	27,17	1,89	21,72	3,74	42,99
A3 OCT in vivo									
Post links = OCT 1.2 501	2,85	4,13	6,03	1,28	30,99	1,9	31,51	3,18	52,74
Post links = OCT 1.2 432	5,10	6,45	8,3	1,35	20,93	1,85	22,29	3,20	38,55
Post links = OCT 1.2 229	1,64	2,33	3,27	0,69	29,61	0,94	28,75	1,63	49,85
A4 OCT in vivo									
RB8.2 378	2,60	4,15	6,89	1,55	37,35	2,74	39,77	4,29	62,26
RB8.2 297	0,71	1,33	2,26	0,62	46,62	0,93	41,15	1,55	68,58
RB8.2 252	0,63	1,11	1,73	0,48	43,24	0,62	35,84	1,10	63,58
RB8.2 219	0,63	0,93	1,41	0,30	32,26	0,48	34,04	0,78	55,32
RB9.2 448	10,08	12,46	15,55	2,38	19,10	3,09	19,87	5,47	35,18
RB9.2 390	14,15	15,75	17,63	1,60	10,16	1,88	10,66	3,48	19,74
RB9.2 136	1,21	1,86	2,78	0,65	34,95	0,92	33,09	1,57	56,47
RB9.1 156	1,06	1,79	2,45	0,73	40,78	0,66	26,94	1,39	56,73
RB9.1 076	0,73	1,45	2,79	0,72	49,66	1,34	48,03	2,06	73,84
RB10.2 0047	2,62	3,37	4,66	0,75	22,26	1,29	27,68	2,04	43,78
RB10.2 0045	2,51	3,72	5,56	1,21	32,53	1,84	33,09	3,05	54,86
RB10.2 0014	1,70	2,19	3,36	0,49	22,37	1,17	34,82	1,66	49,40
A5 OCT in vivo									
ant rechts.2 ref post.1 386	4,96	6,44	8,39	1,48	22,98	1,95	23,24	3,43	40,88
ant rechts.2 ref post.1 384	5,12	6,45	7,48	1,33	20,62	1,03	13,77	2,36	31,55
ant rechts.2 ref post.1 312	4,07	5,69	6,98	1,62	28,47	1,29	18,48	2,91	41,69
ant rechts.2 ref post.1 329	3,95	5,1	6,38	1,15	22,55	1,28	20,06	2,43	38,09
ant rechts.2 ref post.2 154	1,26	2,01	2,92	0,75	37,31	0,91	31,16	1,66	56,85
ant rechts.2 ref post.2 280	1,22	1,73	2,12	0,51	29,48	0,39	18,40	0,90	42,45
post midden=links ref ant.2 467	6,11	7,84	9,83	1,73	22,07	1,99	20,24	3,72	37,84
post midden=links ref ant.2 414	3,69	5,25	7,34	1,56	29,71	2,09	28,47	3,65	49,73
post midden=links ref ant.2 410	3,19	4,6	5,64	1,41	30,65	1,04	18,44	2,45	43,44
Apic lat ref api api.3 432	5,15	6,25	7,35	1,10	17,60	1,1	14,97	2,20	29,93
Apic lat ref api api.3 305	3,07	3,92	5,59	0,85	21,68	1,67	29,87	2,52	45,08
Apic lat ref api api.3 350	3,00	4,07	4,89	1,07	26,29	0,82	16,77	1,89	38,65
Apic lat ref api api.3 392	2,75	3,73	4,71	0,98	26,27	0,98	20,81	1,96	41,61
api api ref api lat.2 460	4,49	5,82	7,04	1,33	22,85	1,22	17,33	2,55	36,22
api api ref api lat.2 359	3,28	4,32	5,58	1,04	24,07	1,26	22,58	2,30	41,22
api api ref api lat.2 216	1,47	2,22	3,89	0,75	33,78	1,67	42,93	2,42	62,21
api api ref api lat.2 251	1,71	2,45	3,44	0,74	30,20	0,99	28,78	1,73	50,29

	PL	Pmuc	Psubmusc	WAmuc	WAmuc(%)	Wasubmuc	WAsubmuc(%	WAt	WAt(%)
A1 histology									
IIIA	1,22	2,75	4,20	1,53	55,64	1,45	34,52	2,98	70,95
IIIB	1,45	3,07	4,75	1,62	52,77	1,68	35,37	3,30	69,47
IIIC	0,79	2,26	3,56	1,47	65,04	1,3	36,52	2,77	77,81
IIID	0,59	1,38	2,27	0,79	57,25	0,89	39,21	1,68	74,01
A2 histology									
IIA	4,02	5,76	6,65	1,74	30,21	0,89	13,38	2,63	39,55
IIB	1,79	3,20	4,13	1,41	44,06	0,93	22,52	2,34	56,66
IIIA	10,80	14,50	19,10	3,7	25,52	4,6	24,08	8,30	43,46
IIIB	3,93	5,72	7,51	1,79	31,29	1,79	23,83	3,58	47,67
VB	4,87	7,59	9,90	2,72	35,84	2,31	23,33	5,03	50,81
A3 histology									
IB	3,00	4,71	5,64	1,71	36,31	0,93	16,49	2,64	46,81
IC	5,20	7,15	8,58	1,95	27,27	1,43	16,67	3,38	39,39
IF	1,49	2,63	3,22	1,14	43,35	0,59	18,32	1,73	53,73
IIA	2,74	4,24	4,98	1,5	35,38	0,74	14,86	2,24	44,98
IIB	1,41	2,38	3,19	0,97	40,76	0,81	25,39	1,78	55,80
IIC	2,33	4,00	4,94	1,67	41,75	0,94	19,03	2,61	52,83
IID	1,26	2,15	3,09	0,89	41,40	0,94	30,42	1,83	59,22
IIE1	1,66	2,45	2,82	0,79	32,24	0,37	13,12	1,16	41,13
IIE2	1,35	2,24	2,68	0,89	39,73	0,44	16,42	1,33	49,63
IIG	1,31	2,04	2,31	0,73	35,78	0,27	11,69	1,00	43,29
A4 histology									
IB	2,96	3,93	4,68	0,97	24,68	0,75	16,03	1,72	36,75
ID	0,23	1,02	1,37	0,79	77,45	0,35	25,55	1,14	83,21
IE	1,05	2,31	2,82	1,26	54,55	0,51	18,09	1,77	62,77
IG	0,16	0,51	0,74	0,35	68,63	0,23	31,08	0,58	78,38
IIIA	9,95	12,32	13,80	2,37	19,24	1,48	10,72	3,85	27,90
IIIB	14,50	16,25	17,10	1,75	10,77	0,85	4,97	2,60	15,20
IIID	0,90	2,39	3,18	1,49	62,34	0,79	24,84	2,28	71,70
IIIF	0,81	1,91	2,31	1,1	57,59	0,4	17,32	1,50	64,94
IIIG	0,49	1,11	1,57	0,62	55,86	0,46	29,30	1,08	68,79
VA	2,02	3,93	4,86	1,91	48,60	0,93	19,14	2,84	58,44
VB	2,85	5,25	6,38	2,4	45,71	1,13	17,71	3,53	55,33
VC	0,58	1,21	1,43	0,63	52,07	0,22	15,38	0,85	59,44
VD	0,62	1,46	1,86	0,84	57,53	0,4	21,51	1,24	66,67
VE	0,66	1,47	1,76	0,81	55,10	0,29	16,48	1,10	62,50
VF	0,90	1,53	1,98	0,63	41,18	0,45	22,73	1,08	54,55
A5 histology									
IA	4,28	5,39	6,87	1,11	20,59	1,48	21,54	2,59	37,70
IB	4,55	5,62	6,46	1,07	19,04	0,84	13,00	1,91	29,57
IC	3,28	4,63	5,57	1,35	29,16	0,94	16,88	2,29	41,11
ID	3,16	4,29	5,68	1,13	26,34	1,39	24,47	2,52	44,37
IE	0,82	1,31	1,58	0,49	37,40	0,27	17,09	0,76	48,10
IF	1,19	1,83	2,39	0,64	34,97	0,56	23,43	1,20	50,21
IIA	6,44	7,31	8,60	0,87	11,90	1,29	15,00	2,16	25,12
IIB	7,37	8,31	8,99	0,94	11,31	0,68	7,56	1,62	18,02
IID	4,21	5,25	5,85	1,04	19,81	0,6	10,26	1,64	28,03
IVB	5,60	7,40	8,36	1,8	24,32	0,96	11,48	2,76	33,01
IVC	4,32	5,69	6,73	1,37	24,08	1,04	15,45	2,41	35,81
IVD	3,60	4,72	5,44	1,12	23,73	0,72	13,24	1,84	33,82
IVE	2,59	3,80	4,71	1,21	31,84	0,91	19,32	2,12	45,01
VA	6,09	7,17	7,99	1,08	15,06	0,82	10,26	1,90	23,78
VB	2,93	3,72	4,28	0,79	21,24	0,56	13,08	1,35	31,54
VC	2,48	3,70	4,19	1,22	32,97	0,49	11,69	1,71	40,81
VD	1,45	1,99	2,21	0,54	27,14	0,22	9,95	0,76	34,39

	PL	Pmuc	Psubmusc	WAmuc	WAmuc(%)	Wasubmuc	WAsubmuc(%	WAt	WAt(%)
A1 OCT ex vivo									
Apicaal 2 hechtingen 1 met naald 240	1,76	2,73	4,06	0,97	35,53	1,33	32,76	2,30	56,65
Apicaal 2 hechtingen 1 met naald 137	1,57	2,62	4,25	1,05	40,08	1,63	38,35	2,68	63,06
Apicaal 2 hechtingen 1 met naald 123	0,94	1,78	4,15	0,84	47,19	2,37	57,11	3,21	77,35
Apicaal 2 hechtingen 1 met naald 99	0,68	1,59	2,20	0,91	57,23	0,61	27,73	1,52	69,09
A2 OCT ex vivo									
Ant ant naald 3 504	3,65	5,62	7,49	1,97	35,05	1,87	24,97	3,84	51,27
Ant ant naald 3 464	1,89	3,41	5,05	1,52	44,57	1,64	32,48	3,16	62,57
Ant post 2 naald 1.1 539	10,45	14,06	19,57	3,61	25,68	5,51	28,16	9,12	46,60
Ant post 2 naald 1.1 487	3,97	5,51	7,75	1,54	27,95	2,24	28,90	3,78	48,77
Post ant naald 1 486	5,79	8,2	12,14	2,41	29,39	3,94	32,45	6,35	52,31
A3 OCT ex vivo									
OCT 1.3 naalden 3 398	3,03	4,88	6,91	1,85	37,91	2,03	29,38	3,88	56,15
OCT 1.3 naalden 3 318	5,45	7,74	9,18	2,29	29,59	1,44	15,69	3,73	40,63
OCT 1.3 naalden 3 136	1,80	3	4,56	1,20	40,00	1,56	34,21	2,76	60,53
OCT 2 rechts 2 naalden.4 481	2,94	4,04	5,84	1,10	27,23	1,8	30,82	2,90	49,66
OCT 2 rechts 2 naalden.4 421	1,66	2,96	4,31	1,30	43,92	1,35	31,32	2,65	61,48
OCT 2 rechts 2 naalden.4 382	2,62	4,64	5,47	2,02	43,53	0,83	15,17	2,85	52,10
OCT 2 rechts 2 naalden.1 292	1,38	2,56	3,51	1,18	46,09	0,95	27,07	2,13	60,68
OCT 2 rechts 2 naalden.3 252	1,66	2,76	4,02	1,10	39,86	1,26	31,34	2,36	58,71
OCT 2 rechts 2 naalden.3 260	1,46	2,7	3,55	1,24	45,93	0,85	23,94	2,09	58,87
OCT 2 rechts 2 naalden.3 116	1,27	2,02	2,68	0,75	37,13	0,66	24,63	1,41	52,61
A4 OCT ex vivo									
LB8 rechts.4 397	2,64	4,3	6,58	1,66	38,60	2,28	34,65	3,94	59,88
LB8 rechts.4 318	1,12	2,46	5,98	1,34	54,47	3,52	58,86	4,86	81,27
LB8 rechts.4 279	0,67	1,23	1,70	0,56	45,53	0,47	27,65	1,03	60,59
LB8 rechts.4 041	0,63	1,03	1,55	0,40	38,83	0,52	33,55	0,92	59,35
LB9 onder.5 hechting laatste verplaatst 459	10,27	12,84	15,99	2,57	20,02	3,15	19,70	5,72	35,77
LB9 onder.5 hechting laatste verplaatst 416	14,49	16,41	19,04	1,92	11,70	2,63	13,81	4,55	23,90
LB9 onder.5 hechting laatste verplaatst 251	1,17	1,85	3,04	0,68	36,76	1,19	39,14	1,87	61,51
LB9 boven.1 353	0,99	1,95	2,21	0,96	49,23	0,26	11,76	1,22	55,20
LB9 boven.1 213	0,84	1,73	2,80	0,89	51,45	1,07	38,21	1,96	70,00
LB10 boven.2 4 naalden 474	2,60	4,09	5,66	1,49	36,43	1,57	27,74	3,06	54,06
LB10 boven.2 4 naalden 425	2,19	3,84	7,05	1,65	42,97	3,21	45,53	4,86	68,94
LB10 boven.2 4 naalden 319	1,21	2,06	2,76	0,85	41,26	0,7	25,36	1,55	56,16
LB10 boven.2 4 naalden 297	0,93	1,72	2,16	0,79	45,93	0,44	20,37	1,23	56,94
LB10 boven.2 4 naalden 258	1,03	1,79	3,05	0,76	42,46	1,26	41,31	2,02	66,23
LB10 boven.2 4 naalden 208	0,90	1,6	2,46	0,70	43,75	0,86	34,96	1,56	63,41
A5 OCT ex vivo									
Ant lat met 3 naalden.3 512	4,74	5,94	7,31	1,20	20,20	1,37	18,74	2,57	35,16
Ant lat met 3 naalden.3 465	5,07	6,38	7,49	1,31	20,53	1,11	14,82	2,42	32,31
Ant lat met 3 naalden.3 456	4,27	5,76	6,99	1,49	25,87	1,23	17,60	2,72	38,91
Ant lat met 3 naalden.3 386	3,99	5,5	6,14	1,51	27,45	0,64	10,42	2,15	35,02
Ant lat met 3 naalden.3 352	1,26	1,92	2,83	0,66	34,38	0,91	32,16	1,57	55,48
Ant lat met 3 naalden.3 324	1,20	2,09	3,32	0,89	42,58	1,23	37,05	2,12	63,86
Post rechts met 3 naald.3 477	5,96	7,24	8,10	1,28	17,68	0,86	10,62	2,14	26,42
Post rechts met 3 naald.3 445	3,61	4,57	5,63	0,96	21,01	1,06	18,83	2,02	35,88
Post rechts met 3 naald.1 412	3,10	4,05	5,12	0,95	23,46	1,07	20,90	2,02	39,45
Api lat 2 naalden.1 472	4,95	6,06	6,80	1,11	18,32	0,74	10,88	1,85	27,21
Api lat 2 naalden.1 431	3,04	3,93	5,02	0,89	22,65	1,09	21,71	1,98	39,44
Api lat 2 naalden.1 332	2,92	3,88	4,46	0,96	24,74	0,58	13,00	1,54	34,53
Api lat 2 naalden.1 303	2,79	3,63	4,35	0,84	23,14	0,72	16,55	1,56	35,86
Api mediaal 2 naalden.4 364	4,60	5,75	6,40	1,15	20,00	0,65	10,16	1,80	28,13
Api mediaal 2 naalden.4 314	3,22	4,28	4,50	1,06	24,77	0,22	4,89	1,28	28,44
Api mediaal 2 naalden.4 246	1,42	2,01	2,45	0,59	29,35	0,44	17,96	1,03	42,04
Api mediaal 2 naalden.4 226	1,69	2,29	2,79	0,60	26,20	0,5	17,92	1,10	39,43

	PL	Pmuc	Psubmusc	WAmuc	WAmuc(%)	Wasubmuc	WAsubmuc(%	WAt	WAt(%)
A1 OCT in vivo									
RB apicaal links tak.1 415	1,63	2,49	3,98	0,86	34,54	1,49	37,44	2,35	59,05
RB apicaal links tak.1 402	1,49	2,37	3,93	0,88	37,13	1,56	39,69	2,44	62,09
RB apicaal links tak.1 283	0,80	1,36	2,80	0,56	41,18	1,44	51,43	2,00	71,43
RB apicaal links tak.1 259	0,68	1,24	2,72	0,56	45,16	1,48	54,41	2,04	75,00
A2 OCT in vivo									
LOK Anterior posterior.6 441	9,93	12,73	17,18	2,80	22,00	4,45	25,90	7,25	42,20
LOK Anterior posterior.6 415	4,37	6,13	7,91	1,76	28,71	1,78	22,50	3,54	44,75
LOK posterior anterior.4 465	4,86	6,86	10,14	2,00	29,15	3,28	32,35	5,28	52,07
A3 OCT in vivo									
Post links = OCT 1.2 501	2,85	4,01	6,09	1,16	28,93	2,08	34,15	3,24	53,20
Post links = OCT 1.2 432	5,03	6,65	10,44	1,62	24,36	3,79	36,30	5,41	51,82
Post links = OCT 1.2 229	1,64	2,42	3,93	0,78	32,23	1,51	38,42	2,29	58,27
A4 OCT in vivo									
RB8.2 378	2,56	4,19	6,02	1,63	38,90	1,83	30,40	3,46	57,48
RB8.2 297	1,03	1,69	2,82	0,66	39,05	1,13	40,07	1,79	63,48
RB8.2 252	0,68	1,27	1,95	0,59	46,46	0,68	34,87	1,27	65,13
RB8.2 219	0,66	1,12	1,60	0,46	41,07	0,48	30,00	0,94	58,75
RB9.2 448	10,24	12,78	15,52	2,54	19,87	2,74	17,65	5,28	34,02
RB9.2 390	14,25	16,57	18,48	2,32	14,00	1,91	10,34	4,23	22,89
RB9.2 136	1,18	1,85	3,48	0,67	36,22	1,63	46,84	2,30	66,09
RB9.1 156	1,03	1,83	2,38	0,80	43,72	0,55	23,11	1,35	56,72
RB9.1 076	0,81	1,68	3,04	0,87	51,79	1,36	44,74	2,23	73,36
RB10.2 0047	2,65	3,86	5,01	1,21	31,35	1,15	22,95	2,36	47,11
RB10.2 0045	2,51	3,94	6,39	1,43	36,29	2,45	38,34	3,88	60,72
RB10.2 0014	1,62	2,57	3,52	0,95	36,96	0,95	26,99	1,90	53,98
A5 OCT in vivo									
ant rechts.2 ref post.1 386	4,85	6,25	7,86	1,40	22,40	1,61	20,48	3,01	38,30
ant rechts.2 ref post.1 384	5,05	6,36	7,84	1,31	20,60	1,48	18,88	2,79	35,59
ant rechts.2 ref post.1 312	4,21	5,6	6,70	1,39	24,82	1,1	16,42	2,49	37,16
ant rechts.2 ref post.1 329	3,95	5,25	6,25	1,30	24,76	1	16,00	2,30	36,80
ant rechts.2 ref post.2 154	1,27	2,11	3,15	0,84	39,81	1,04	33,02	1,88	59,68
ant rechts.2 ref post.2 280	1,22	1,75	2,42	0,53	30,29	0,67	27,69	1,20	49,59
post midden=links ref ant.2 467	6,23	8,21	10,50	1,98	24,12	2,29	21,81	4,27	40,67
post midden=links ref ant.2 414	3,64	5,28	6,54	1,64	31,06	1,26	19,27	2,90	44,34
post midden=links ref ant.2 410	3,11	4,77	6,00	1,66	34,80	1,23	20,50	2,89	48,17
Apic lat ref api api.3 432	4,93	6,75	9,31	1,82	26,96	2,56	27,50	4,38	47,05
Apic lat ref api api.3 305	3,07	4,16	5,48	1,09	26,20	1,32	24,09	2,41	43,98
Apic lat ref api api.3 350	2,96	4,08	4,92	1,12	27,45	0,84	17,07	1,96	39,84
Apic lat ref api api.3 392	2,75	3,78	4,79	1,03	27,25	1,01	21,09	2,04	42,59
api api ref api lat.2 460	4,59	5,86	7,42	1,27	21,67	1,56	21,02	2,83	38,14
api api ref api lat.2 359	3,23	4,36	5,18	1,13	25,92	0,82	15,83	1,95	37,64
api api ref api lat.2 216	1,44	2,29	4,31	0,85	37,12	2,02	46,87	2,87	66,59
api api ref api lat.2 251	1,67	2,52	2,91	0,85	33,73	0,39	13,40	1,24	42,61
